# Collectanea Medica: Consisting of Anecdotes, Facts, Extracts, Illustrations, &c.

**Published:** 1822-02

**Authors:** 


### COLLECTANEA MEDICA:

CONSISTING OF

### ANECDOTES, FACTS, EXTRACTS, ILLUSTRATIONS, &c.

*Relating to the History or the Art of Medicine, and the Collateral Sciences.*

Floriferis, ut apes, in saltibus omnia libant,

Omnia nos, itidem, depascimur aurea dicta.

---

ART. I.—*An Address, delivered before the Medical Society of New-York, at their Anniversary Meeting, at the Capitol, in the City of Albany, February* 6, 1821, *on the Functions and Diseases of the Liver.* By JOHN STEARNS, M.D. President of the Society.

[Concluded from page 47.]

A FEW remarks will now be bestowed upon those organs in their diseased state, which are affected by the morbid condition of the liver, and which are associated with it by the influence of a mutual sympathy.

1st. *The Spleen.*—The subserviency of the spleen to the liver, and their mutual connexion, are further evinced in the diseases which the morbid affection of one produces upon the other. Dissections show that an indurated and contracted liver precedes an enlarged spleen. This has been explained by Morgagni and others, by ascribing, to the contracted and unyielding vessels of the liver, an obstacle to the usual flow of blood from the spleen, while an increasing quantity continues to enter it by the splenic artery. An accumulation of blood, and the consequent enlargement of that viscus, become the necessary result.

The same author found an enlarged liver generally to accompany an enlarged spleen; which has been confirmed by more modern dissectors.

It was a common opinion among the ancients, that a diseased liver always preceded dropsy, and that a morbid state of the spleen soon afterwards succeeded. Although modern dissections do not confirm the order of this affection, yet, in every case of established ascites, one of these viscera has been found diseased. It was for this reason that Albertini considered this species of dropsy incurable.

2d. *The Stomach.*—This has been already alluded to, in explaining the associate connexion between digestion and the secretion of bile: and, although imperfect digestion has generally been considered as the precursor of vitiated bile, there are strong reasons for believing that the latter is more generally the exciting cause of the former.

As the sensibility of the liver exceeds that of the stomach, it is consequently more liable to become affected by disease. Hence hepatitis is more frequent than gastritis; and also that peculiar depression of mind, so strongly indicative of morbid biliary secretion, generally precedes an impaired state of the digestive organs. It is for the same reason that some writers have assigned to the liver the power of transferring diseases of the stomach to other parts which have more sensibility, and also to the minor glands, which derive their diseases from this, their parental gland, as children inherit the morbid taint of their ancestors.

3d. *The Skin.*—The hepatic sympathy with the skin is conspicuous and extensive, and, although well known to the profession, has not received that due notice, in the history and treatment of hepatic diseases, which its importance requires. Erysipelas,—a certain species of phlegmon on the extremities,—various cutaneous eruptions,—and a peculiar redness of the nose, are well-known indications of a diseased liver, and of the reciprocal influence of this sympathy. It is further evinced by the same causes producing a cotemporaneous secretion of bile and of perspiration. This has been particularly noticed by Dr. Johnson, in the tropical climates of the east. The extreme heat of that climate so violently excited the minute vessels of the skin and liver, as to procure, at the same time, copious discharges of bile and of perspiration. To obviate the debilitating effects, and to moderate the excess of these discharges, the Asiatic and the African are compelled to anoint their skin with oil. It is thereby defended against the excessive heat; and the immoderate flow of bile and of perspiration is equally restrained. This also prevents that torpor of the skin and liver, so common in those climates, which succeeds to their inordinate action, or to their subsequent exposure to wet and cold. By this torpor both secretions are diminished, and the bile accumulates in the liver, where it becomes vitiated by retention, and ultimately produces painful effects upon the alimentary canal.

Those who have indurated livers, sallow complexions, torpid bowels, and a paucity of bilious secretion, have also a dry, harsh, and contracted skin. Both of these organs are also similarly affected by the depressing passions of fear and grief.

In diabetes, chlorosis, mania, and dysentery, perspiration is always deficient, and the secretion of bile also evidently diminished. Doctors Balfour and Johnson confirm this opinion in dysentery; and add, that if these secretions can be restored, and the plethora of the portal circle reduced, before the intestines are much injured, the cure will be completed.

Contrary to common opinion, cholera affords another instance of diminished bile and a dry skin. When the bile is checked, and the vessels on the surface constricted, in the incipient stage of this disease, if the efforts to vomit, which immediately ensue, do not succeed in restoring perspiration and the secretion of bile, death soon occurs. The deluges of bile, which burst forth when secretion is restored, show the great plethora in the portal vessels which its temporary suppression had induced.

The warm bath, stimulating cordials, opium, calomel, frictions and hot applications to the skin, cure cholera, by restoring the hepatic and cutaneous secretions. And I have no hesitation to affirm, that frequent ablutions, frictions, and other suitable purifications and incitements to the skin, will generally counteract a strong predisposition to hepatic diseases.

4th. *The Mind.*—The influence reciprocated between the mind and the liver, is productive of well-known and important effects. Violent anger affects the liver with spasm, and causes a regurgitation of bile into the stomach, and immediate vomiting.

Hepatic diseases are generally accompanied with a depression of spirits, a tædium vitæ, melancholy, hypochondriasis, and a choleric disposition. It is, therefore, on a healthy state of this organ that our happiness in this life essentially depends. A cheerful disposition is the most effectual antidote to a diseased liver; and I can attest to the cure of one of the most inveterate and dangerous affections of this organ, by the uniform preservation of a remarkably cheerful mind, continued in opposition to the influence of the disease, by a succession of very cheering occurrences.

The idiopathic diseases of the liver may be reduced to the following organic affections:

I. Inflammation. This may affect the membranes or the substance of this gland; and is acute when it is confined to the hepatic arteries, or chronic, when it affects only the venous or secreting system. The first is often mistaken for an inflammatory affection of the chest, the stomach, and contiguous muscles; and the latter, for dyspepsy. This may terminate in resolution, scirrhus, suppuration, or gangrene.

II. Tubercles. These consist of the following species:

1st. Common tubercles, which extend through the whole substance of the liver, and are generally caused by intemperance.

2d. Large white tubercles, which constitute its scirrhosity.

3d. Soft brown tubercles, which are incorrectly denominated scrofulous.

4th. Scrofulous tumors, which nearly resemble the tubercles of the lungs.

III. A soft state of the substance of the liver, which John Hunter denominated interstitial absorption. It seldom affects the young, but is a common disease of advanced life.

IV. The Induration of the Liver. This is a very common disease.

V. Hydatids. These are common, and, when in the substance of the liver, are found enclosed in a cyst; but, when on the surface, hang pendulous into the abdomen. They are said to contain animals imperfectly formed.

VI. Increased secretion of bile.

VII. Diminished secretion of bile.

VIII. Morbid secretion of imperfect bile.

The gall-bladder is also liable to—

I. Inflammation. This often produces adhesion of its coats to the contiguous parts, but seldom ulceration.

II. Scirrhosity.

III. Ossification of its coats.

The hepatic and cystic ducts are also liable to become obliterated, and are often dilated by the passage of the gall-stones.

The bile is diseased when it is imperfectly secreted, and thence becomes extremely acrid to the intestines, or is ejected from the stomach with the usual appearances of black vomit; or when, by long stagnation in the gall-bladder, it becomes green and viscid.

The symptoms and treatment of the diseases of the liver are so minutely detailed and judiciously described by practical writers, that I am compelled, by the limits of this address, to refer to them, for a correct knowledge of the diagnostics and general mode of cure. I cannot, however, refrain from observing, that calomel constitutes a very important remedy in most hepatic affections. It relieves torpor and inflammation, opens obstructions, and promotes the secretion of bile. Its successful operation is materially aided by the application of epispastics and mercurial ointment to the right hypochondriac region. The sympathy between the skin and liver, before alluded to, designates the importance of these auxiliaries to the surface. When these remedies have succeeded in restoring the healthy action of the liver, and the consequent secretion of bile, the peristaltic motion of the intestines is immediately excited,—the previous constipation of the bowels begins to yield,—the evacuations become regular, and exhibit the appearance of bile in each discharge. With the inceptive appearance of these favourable effects, we discover an abatement of all the symptoms of the disease, with an increased secretion of bile and of perspiration.

These indications evince the necessity of a total change in the previous course of treatment. It must be obvious, to every practitioner of experience, that a perseverance in the same remedies would excite the liver into preternatural action, and thus accelerate the secretion of morbid bile; which, by its acrid qualities, would produce an obstinate diarrhœa, that no remedies could restrain while these stimulants were continued.

The skill of the physician is not more eminently displayed in the selection of his remedies, than in the adaptation of suitable doses to varying gradations of disease, or in his sagacity to discover the precise moment when to arrest the course of treatment. To persist in it beyond this period, however salutary and successful in the commencement, would constitute it the source of a new disease, that would baffle the skill of the physician, disappoint the elated hopes of the patient, and produce despondency and death.

Errors in practice, from this cause, have prejudiced the inconsiderate against some of the most valuable articles of the materia medica, which their ignorance and obstinacy have finally succeeded in consigning to oblivion.

---

Art. II.—*On some remarkable Deviations in the Natural Distribution of Blood-Vessels.* By J. F. Meckel.—(Deutsches Archives für die Physiologie. Sechster band. dr. heft.)

In the course of last winter, I had occasion to notice certain remarkable deviations in the distribution of blood-vessels in the body of a male child, who died at nine years of age.

The most singular of these deviations, and one most rarely to be met with, consisted in the inferior extremity of the pectoral aorta giving out, half an inch from where it penetrated the diaphragm, a considerable vessel, a third of an inch in diameter, and an inch and a half long, describing an arch, the convexity of which was turned to the right of the chest, and stretching itself as far as the external border of the inferior surface of the left lung. There it divided itself into two principal branches, of which the internal, rising almost perpendicularly, ramified itself over the internal surface of the inferior lobe of the lung; whilst the external, which took an oblique direction, distributed its various branches to the external surface of the same lobe. This accessory vessel served to supply the blood to that organ alone. There was no corresponding branch on the right side of the chest; and the vessel, of which I have just given a description, did not send off any branches to the right lung. There were veins corresponding to the blood-vessel in question, which terminated in the inferior left pulmonary vein.

No other deviation worthy of notice was found in this stage of the dissection. The pulmonary artery and the aorta, as well as the pulmonary veins and vena cavæ, retained their ordinary calibre; although the accessory pulmonary artery greatly contributed to complete the functions of the left branch of the ordinary artery of the same denomination.

The bronchial vessels were arranged as usual.

Nothing particular was observed respecting the heart and lungs, except that the inferior lobe of the left lung offered, forwards and upwards, a small appendix, forming a middle lobe, which received its blood-vessels from the ordinary pulmonary artery; while the middle lobe of the right side was somewhat more separated from the others, than in ordinary cases.

The other viscera were, in every respect, perfect, and presented no pathological derangement.

Hitherto there have been only two other cases which bear, at all, any resemblance to this; and of those I have given a complete history in my Manual of Pathological Anatomy. The one was described by Huber, the other by Mangars. Mine, however, differs from both in a notable manner.

I was not able to procure any information relative to the physiological condition of the child.

Besides the above curious facts, several aberrations, with regard to the distribution and ramification of the humeral and brachial blood-vessels, were observed in the same individual.

---

### Art. III.—*A second Table of Suicides, taken from the Work of* Mr. Fournier, *intitled "Recherches Statistiques sur la Ville de Paris."*

Table of Suicides, *committed or attempted, in the Department of the Seine, during the Year* 1818.

| Suicides | | |
|---|---|---|
| Followed by death, 241 | Total of Suicides, 330 | Not followed by death, 89 |

| Effected or attempted, | | | | |
|---|---|---|---|---|
| By men, 192 | By women, 138 | Total, 330 | By batchelors, 165 | By married persons, 165 |

| *Means of Destruction employed.* | *No. of Suicides.* | *Total.* | *No. of Suicides.* | *Presumed Motives for the Suicide.* |
|---|---|---|---|---|
| Voluntary falls | 40 | 330 | 19 | Love. |
| Strangulation | 27 | | 151 | Diseases, hypochondriasis, weakness, mental alienation, domestic quarrels, and grief. |
| Cutting or other instruments | 28 | | 45 | Bad conduct, gaming, lottery, debauchery, &c. |
| Fire-arms | 48 | | 56 | Poverty, loss of places or employments, derangement of affairs. |
| Poison | 21 | | 8 | Dread of reproaches and punishments. |
| Suffocation by the vapour of charcoal | 35 | | 50 | Motives unknown. |
| Do. by submersion | 131 | | | |

In our department of Medical Intelligence, we have inserted the Bill of Mortality in London, and adjoining villages, for the year 1821, from which it appears, that the number of suicides during that period, in a population of more than a million of inhabitants, amounted to thirty-two only; presenting a singular contrast, when compared to the number of suicides committed, in the course of twelve months, in the department of the Seine, as appears manifest from the preceding table, and from that which we inserted in the last Number. Indeed, since the Revolution, the French seem more prone to self-destruction than any other nation; and, with regard to the means employed for carrying their purpose into effect, they have also far outstripped the ingenuity of foreign people. We have had frequent opportunities of investigating this subject during our long residence in Paris; and we recollect hearing it stated, by two professors on medical jurisprudence, Orfila and Royer-Collard, that, in some of the printed cotton manufactories, workmen, not unfrequently, attempted to destroy themselves by taking enemas of a solution of indigo in sulphuric acid, as a preparation with which they had become familiarized from the nature of their work, and of which they well knew the pernicious and fatal effects. These attempts proved, generally, effectual. Soon after the unfortunate son of the present Count Berthollet, the patriarch of French chemists, put an end to his existence by shutting himself up in a small cabinet, in which he had previously lighted a charcoal fire, so as to impregnate the atmosphere with the noxious gas, numerous examples of a similar species of suicide were recorded, not only in the capital, but throughout France; a circumstance which Mr. Royer Collard seemed inclined to attribute to the process resorted to by young Berthollet having been made generally known, through the public journals.

